# Optimization of radiotherapy for neck carcinoma metastasis from unknown primary sites: a meta-analysis

**DOI:** 10.18632/oncotarget.12852

**Published:** 2016-10-24

**Authors:** Xiaomei Liu, Dianhe Li, Na Li, Xiaoxia Zhu

**Affiliations:** ^1^ Department of Radiation Oncology, Nanfang Hospital, Southern Medical University, Guangzhou 510515, China

**Keywords:** neck carcinoma metastasis, unknown primary, NCUP, radiotherapy, meta-analysis

## Abstract

This meta-analysis was designed to evaluate radiotherapy (RT) options preferable for neck cancer metastases from unknown primary sites (NCUP). Relevant articles published up through September 2015 were selected from EMBASE, Cochrane, PubMed and Web of Science. Thirty-three articles were identified, and relative risks (RRs) and 95% CIs for all pre-specified endpoints were calculated. Surgery plus RT showed an advantage for 5-year overall survival (OS) (RR 0.66, 95% CI 0.52–0.83, *p* = 0.0004) and neck recurrence (NR) (RR = 0.74, 95% CI 0.59–0.92, *p* = 0.008) compared to RT alone. The RRs for NR, primary tumor emergence (PTE), and 5-year disease free survival (DFS) for bilateral neck compared to ipsilateral neck irradiation were 0.61 (95% CI 0.41–0.91, *p* = 0.01), 0.44(95% CI 0.26–0.77, *p* = 0.004), and 0.81 (95% CI 0.64–1.03, *p* = 0.09), respectively. Irradiation of the neck plus potential primary tumor sites (PPTS) showed a benefit for 5-year DFS (RR 0.75, 95% CI 0.61–0.92, *p* = 0.005), NR (RR = 0.72, 95% CI 0.56–0.92, *p* = 0.009), and PTE (RR = 0.23, 95% CI 0.12–0.45, *p* < 0.0001) compared to neck-only irradiation. Adverse events occurred more frequently with bilateral neck plus PPTS irradiation. For NCUP, surgery plus RT of the bilateral neck and PPTS was associated with greater improvement of clinical outcomes.

## INTRODUCTION

Neck cancer metastasis with an unknown primary site (NCUP) presents in patients with neck lymph node involvement in the absence of an identifiable primary tumor [1–3]. The histopathology of NCUP consists of squamous cell carcinoma, adenocarcinoma, and other undifferentiated carcinomas [1–3]. The often-extensive diagnostic workup to identify the primary site can include physical examination, chest X-ray, endoscopy, biopsy, computed tomography (CT) or magnetic resonance imaging (MRI), and positron emission tomography (PET). Nonetheless, in approximately 2% to10 % of NCUP cases the primary site remains unidentified [1–12].

NCUP is thought to be potentially curable [2], but the data addressing the therapeutic protocols and outcomes of NCUP treatment are limited and controversial. The proposed treatment modalities include surgery alone, radiotherapy (RT) alone, and a combination of RT and surgery. Opinions on the field design for RT also vary. Some investigators have recommended involved-field irradiation, such as ipsilateral neck irradiation only [7, 11–14], while others suggest extended field irradiation, including prophylactic irradiation of potential head and neck mucosal sites and both sides of the neck [1, 4, 6, 15]. Differences in treatment strategy and patient selection have led to inconsistent results. Consequently, the reported 5-year overall survival (OS) rates for patients with NCUP range from 16% to 86%, and the local control rates range from 37% to 91%. The present meta-analysis was performed in an effort to identify the optimal treatment regimen for NCUP, focusing in particular on the optimal way to schedule RT.

## RESULTS

### Description of selected studies and quality assessment

**Figure 1:**
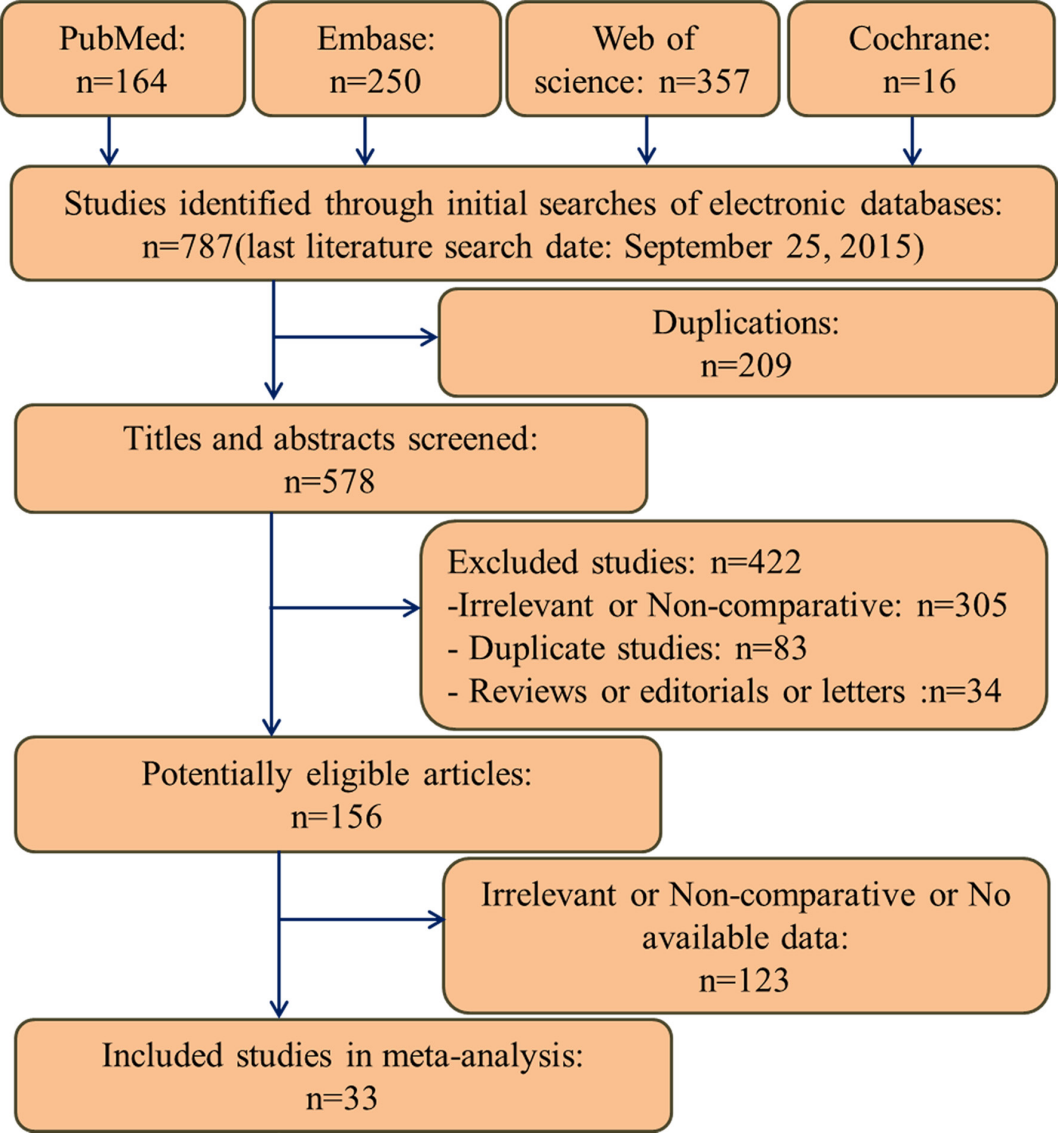
Flow diagram of studies identification and selection

A total of 787 articles were identified, of which 33 articles qualified for inclusion. The flow diagram for study selection is shown in Figure 1. The characteristics of the included studies are summarized in Tables 1 and 2. Although methods for managing missing data are not adequately described in some studies, none of the included studies had a NOS < 6, which suggests all were of high quality.

**Table 1:** Baseline characteristics of the included studies

Study	Patients No.	Age(year)	Studyperiod	Country	Follow up	Gender(Male/Female)	Design	Quality score	Histology	*Nodal stage (NX-1/N2-3)	Nodal level (I-II/III-IV)
1973Jesse	184	NA	1948–1968	USA	> 3Y	NA	R	7	1,3	63/121	162/48
1975Fried	43	NA	1958–1969	USA	NA	2:1	R	6	1,2,3	NA	NA
1979Nordstrom	39	60(13–88)	1960–1973	USA	NA	40/11	R	6	1,2,3	8/43	NA
1981Leipzig	32	51(22–76)	1969–1977	USA	NA	2:1	R	7	1	14/18	NA
1986Carlson	93	NA	1968–1980	USA	> 3Y	70/23	R	7	1,2,3	41/52	61/32
1987Bataini	138	57.5(15–82)	1960–1980	USA	> 5Y	117/21	R	8	1	45/93	NA
1990Glynne	58	61(12–85)	1954–1986	UK	35(4–300)M	53/34	R	7	1,3	11/76	NA
1990Harper	69	58.5(26–91)	1964–1986	Australia	> 2Y	58/11	R	8	1	18/51	NA
1990Marcial	72	58.5(24–85)	1965–1987	USA	NA	50/22	R	6	1,3	12/60	35/37
1995Weir	144	60(28–90)	1970–1986	Canada	3.5(4–15)Y	110/34	R	8	1,2,3	79/65	NA
1997Reddy	52	59(30–79)	1974–1989	USA	> 5Y	50/2	R	9	1	9/43	NA
1997 Sinnathamby	67	62(29–84)	1983–1992	Australia	7M-7Y	51/18	R	8	1,3	9/60	NA
1997van der	44	66(20–86)	1974–1991	Netherlands	7.3Y(2–18.8)	35/9	R	7	1,2,3	4/40	NA
1998Colletier	136	59(25–83)	1968–1992	Canada	58(3–267)M	103/33	R	7	1	41/95	104/32
1998Strojan	56	56(33–81)	1975–1994	Slovenia	8.6(1.6–17.8)Y	50/6	R	8	1	6/50	39/17
2000Grau	352	62(18–92)	1975–1995	Denmark	5Y	248/104	R	7	1,3	48/225	277/0
2000McMahon	34	67(45–84)	1987–1998	Australia	2.7(0.9–6.7)Y	28/10	R	8	1	6/32	31/7
2002Tong	45	57(29–91)	1988–1998	Hong Kong	79M(27–110)	37/8	R	7	1,2,3	7/38	20/25
2002Yalin	114	48(18–78)	1976–1988	China	NA	NA	R	7	1,2,3	33/81	NA
2002Zuur	14	62.6(34.7–82.8)	1975–1999	Netherlands	2–67M	8/8	R	7	1,2	2/13	3/12
2006Boscolo	79	64.7±9.3	1980–2001	Italy	15Y	69/13	R	8	1	10/62	60/12
2007Aslani	61	57(37–87)	1987–2002	Canada	31(7–168)M	49/12	R	7	1,3	16/45	53/8
2007Beldi	113	59.3(23–88)	1980–2004	Italy	NA	93/20	R	7	1,2,3	21/92	83/30
2008Huang	31	63.3(36–84)	1980–2000	Taiwan	NA	35/13	R	8	1	3/45	39/9
2009Ligey	95	59(38–80)	1990–2007	France	3.3Y(5M-11.7Y)	84/11	R	7	1,3	25/70	77/18
2009Lu	60	53(23–81)	1989–2003	China	58(10–135)M	46/14	R	8	1	10/50	44/16
2009Rodel	58	55(37–77)	1980–2004	Germany	83.5M(24–162)	58/10	R	7	1,2,3	9/49	55/3
2011Chen	60	60(42–90)	2001–2009	USA	30M(3–90)	39/21	R	8	1	5/55	51/9
2011Wallace	179	61(26–89)	1990–2006	USA	4.2Y(0.2–25.4)	157/22	R	8	1	18/161	NA
2012Fakhrian	65	60(39–90)	1988–2009	Germany	64(3–219)M	52/13	R	9	1,3	14/51	46/19
2012Perkins	46	60(40–82)	1989–2008	USA	4.6Y(7M-18Y)	NA	R	9	1	3/43	NA
2014Demiroz	41	53(38-72)	1994-2009	USA	11-126M	37/4	R	9	1	4/37	33/8
2015Straetmans	46	NA	1997-2010	Netherlands, Germany	NA	44/7	R	8	1	4/42	NA

* According to UICC/AJCC staging system. R = retrospective; Y = year; M = month; NA = Not available;

1 = squamous cell carcinoma; 2 = adenocarcinoma; 3 = undifferentiated carcinoma

**Table 2:** Baseline characteristics of the treatments of the included studies

Study	Patients No.	Chemotherapy	Radiotherapy		Surgery	
		Patients No.	Patients No.	Treatment volume	Patients No.	Types of surgery
1973Jesse	184	0	80	BN+PPTS,IN+PPTS,BN,IN	132	ND,E
1975Fried	43	0	43	BN+PPTS,IN+PPTS,BN,IN	16	ND
1979Nordstrom	39	0	39	BN+PPTS,IN+PPTS,IN	34	ND
1981Leipzig	32	0	24	BN+PPTS,IN+PPTS,BN,IN	24	ND
1986Carlson	93	0	93	BN+PPTS,IN+PPTS,BN,IN	70	ND,E
1987Bataini	138	0	138	BN+PPTS,BN,IN	48	ND
1990Glynne	58	1	58	BN+PPTS,IN+PPTS,BN,IN	6	ND
1990Harper	69	0	69	BN+PPTS,IN+PPTS,BN,IN	30	ND
1990Marcial	72	0	72	BN+PPTS,IN+PPTS,BN,IN	31	ND,E
1995Weir	144	0	144	BN+PPTS,IN+PPTS,BN,IN	71	E
1997Reddy	52	0	52	BN+PPTS,IN	39	ND,E
1997Sinnathamby	67	0	63	BN,IN	44	ND,E
1997van der	44	0	44	BN+PPTS,IN+PPTS,BN,IN	31	ND,E
1998Colletier	136	0	136	BN+PPTS,IN+PPTS,BN,IN	136	ND,E
1998Strojan	56	0	56	BN+PPTS,IN+PPTS,IN	56	ND,E
2000Grau	352	0	250	BN+PPTS,IN	169	ND,E
2000McMahon	34	0	34	BN+PPTS,IN	34	ND
2002Tong	45	8	36	BN+PPTS	13	ND,E
2002Yalin	114	39	76	BN+PPTS,IN+PPTS,BN,IN	24	ND,E,T
2002Zuur	14	0	14	BN+PPTS,IN+PPTS,BN,IN	7	ND
2006Boscolo	79	0	79	BN+PPTS,IN	50	ND
2007Aslani	61	0	61	BN+PPTS,BN,IN	29	ND,E
2007Beldi	113	21	113	BN+PPTS,BN,IN	99	ND,E
2008Huang	31	16	31	BN+PPTS	30	ND
2009Ligey	95	43	95	BN+PPTS,IN+PPTS,BN,IN	79	ND
2009Lu	60	14	60	BN+PPTS,BN,IN	9	E
2009Rodel	58	22	50	BN+PPTS,IN	53	ND
2011Chen	60	32	60	BN+PPTS,IN+PPTS,IN	45	ND,E
2011Wallace	179	13	179	BN+PPTS,IN+PPTS,IN	109	ND
2012Fakhrian	65	19	65	BN+PPTS,IN	51	ND,E
2012Perkins	46	14	46	BN+PPTS,IN+PPTS,BN,IN	40	ND,E
2014Demiroz	41	25	41	BN+PPTS,BN,IN	22	ND
2015Straetmans	51	8	48	BN+PPTS,BN,IN	51	ND

Abbreviations: BN = Bilateral neck; IN = Ipsilateral neck; PPTS = Potential primary tumor sites.

Neck dissection = ND, Excision = E, Thyroidectomy = T.

### RT alone versus RT combined with surgery

**Figure 2:**
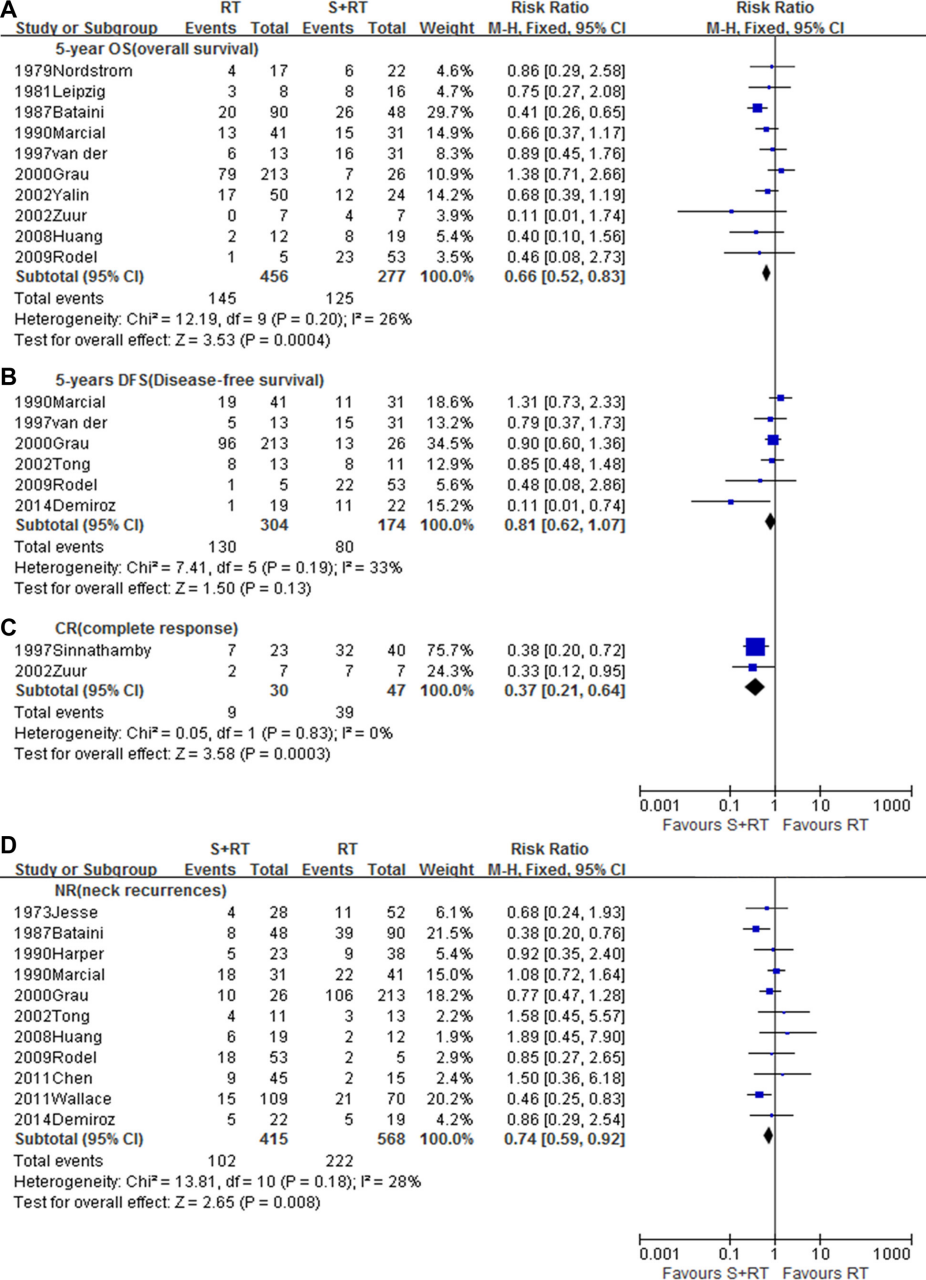
Meta-analysis of (**A**) 5-year OS, (**B**) 5-year DFS,(**C**) CR, (**D**) NR between RT alone and RT combined with surgery. S, Surgery; RT, Radiotherapy.

Eighteen studies [2, 5, 9, 10, 16–29], with a total of 1582 patients, were included in this analysis. Compared to RT alone, the combination of RT and surgery significantly improved 5-year OS (RR = 0.66, 95% CI 0.52–0.83, *p* = 0.0004) (Figure 2A). The benefit to 5-year DFS showed a similar trend (RR = 0.81, 95% CI 0.62–1.07, *p* = 0.13) (Figure 2B), though this did not reach statistical significance. Additionally, surgery in combination with RT was associated with a significantly decreased NR rate (RR = 0.74, 95% CI 0.59–0.72, *p* = 0.008) (Figure 2D) and an increased CR rate (RR = 0.37, 95% CI 0.21–0.64, *p* = 0.0003) (Figure 2C).

### Ipsilateral versus bilateral neck irradiation

**Figure 3:**
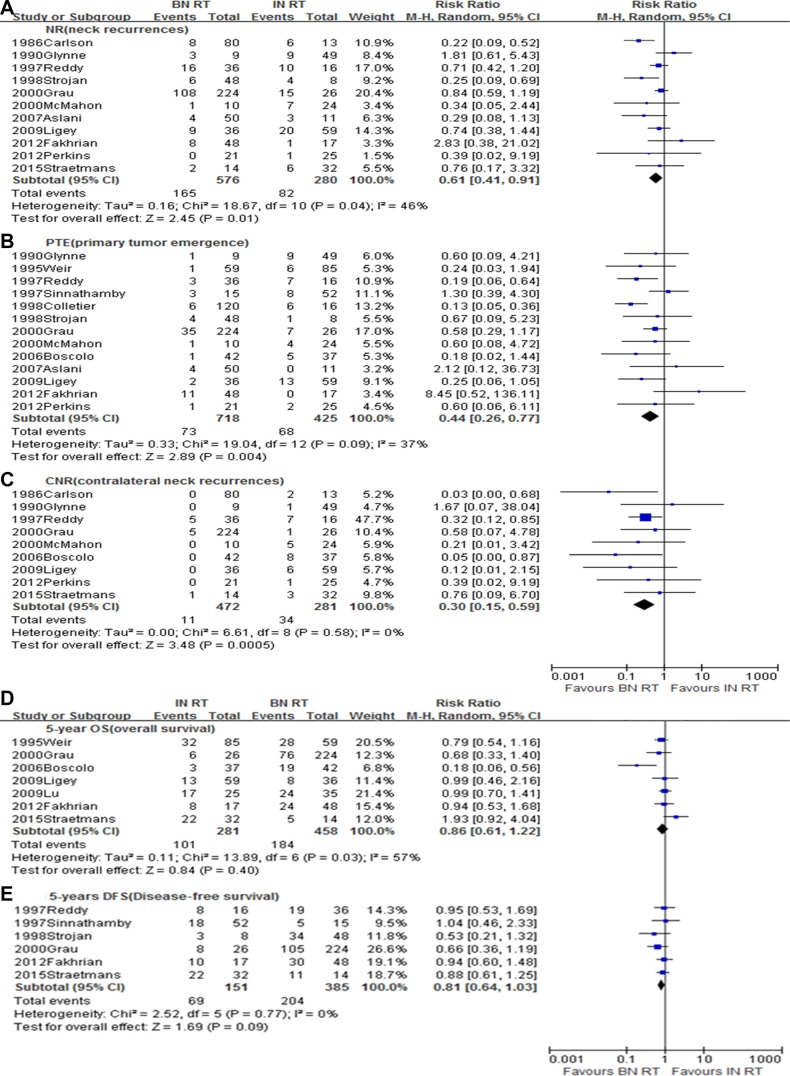
Meta-analysis of (**A**) NR, (**B**) PTE, (**C**) CNR, (**D**) 5-year OS, and (**E**) 5-year DFS between ipsilateral neck irradiation and bilateral neck irradiation. BN, Bilateral neck; IN, Ipsilateral neck; RT, Radiotherapy.

Sixteen studies [1, 3–7, 11–15, 23, 20–33] with a total of 1449 patients, meeting the inclusion criteria were selected for this analysis (Table B). Bilateral neck irradiation contributed to better local control with a lower NR rate (RR = 0.61, 95% CI 0.41–0.91, *p* = 0.01) (Figure 3A) and PTE rate (RR = 0.44, 95% CI 0.26–0.77, *p* = 0.004) (Figure 3B). This was especially true for contralateral neck control (RR = 0.30, 95% CI 0.15–0.59, *p* < 0.0005) (Figure 3C). Moreover, when compared to ipsilateral irradiation, bilateral neck irradiation showed a potential survival advantage with greater 5-year OS (RR = 0.86, 95% CI 0.61–1.22, *p* = 0.40) (Figure 3D) and 5-year DFS (RR = 0.81, 95% CI 0.64–1.03, *p* = 0.09) (Figure 3E), although this benefit was not statistically significant.

### Neck-only versus neck plus PPTS irradiation

**Figure 4:**
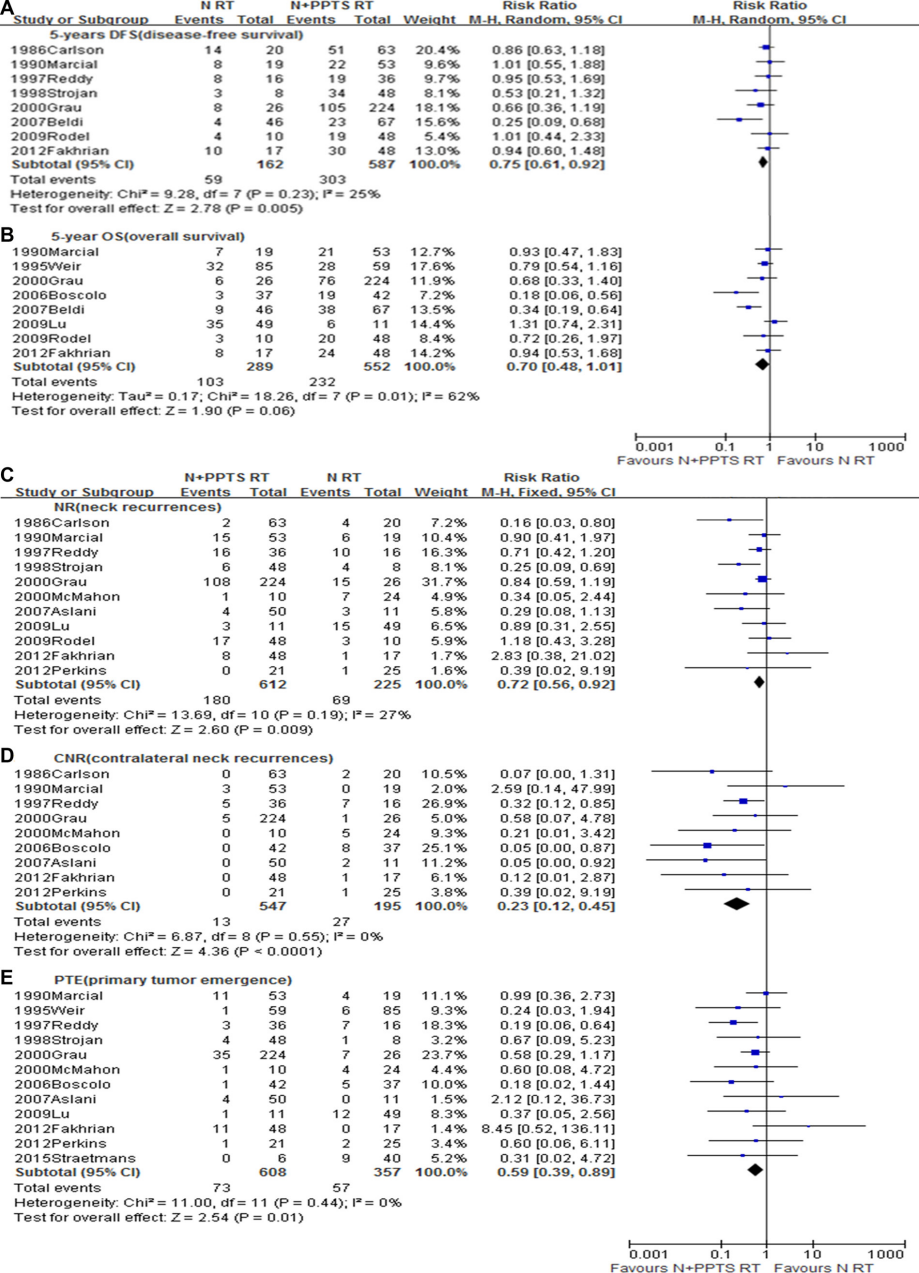
Meta-analysis of (**A**) NR, (**B**) PTE, (**C**) CNR, (**D**) 5-year OS, and (**E**) 5-year DFS between neck only irradiation and neck plus potential primary tumour sites irradiation. N, Neck only; N+PPTS, Neck plus potential primary mucosa sites; RT, Radiotherapy.

Fifteen studies [1–8, 11–13, 15, 22, 30, 33], with a total of 1347 patients, fulfilled the inclusion criteria and were included in this analysis. As compared to neck-only irradiation, superior 5-year DFS (RR 0.75, 95% CI 0.61–0.92, *p* = 0.005) (Figure 4A) and a trend toward increased 5-year OS (RR = 0.70, 95% CI 0.48–1.01, *p* = 0.06) (Figure 4B) were observed in patients treated with neck and PPTS irradiation. In addition, neck and PPTS irradiation had a significant advantage for reducing NR rate (RR = 0.72, 95% CI 0.56–0.92, *p* = 0.009) (Figure 4C), CNR rate (RR = 0.23, 95% CI 0.12–0.45, *p* < 0.0001) (Figure 4D) and PTE rate (RR = 0.59, 95% CI 0.39–0.89, *p* = 0.01) (Figure 4E).

### Toxic effects of different radiotherapeutic regimens

Only two of the included studies [11, 15] compared toxicities between ipsilateral neck irradiation and bilateral neck plus PPTS irradiation, and the toxicity data could only be assessed for severe acute toxicity and xerostomia. We found there was an increased risk of severe acute toxicity (RR = 1.91, 95% CI 1.26–2.88, *p* = 0.002) (Supplementary Figure S1A) and xerostomia (RR = 6.82, 95% CI 0.96–48.55, *p* = 0.06) (Supplementary Figure S1B) in the group with bilateral neck and PPTS irradiation.

## DISCUSSION

Optimal treatment for patients with NCUP remains uncertain. The incidence of NCUP is about 3 cases per 1,000,000 per year. Its rarity makes randomized and prospective studies unavailable, and leaves clinicians with only small retrospective studies for clinical decision making. To the best of our knowledge, this study is the first meta-analysis with a focus on comparing the therapeutic efficacies of different treatment regimens, and on providing a higher level of evidence for optimizing the RT schedule in NCUP.

Some studies held that surgery plus RT resulted in a higher probability of cure [2, 5, 9, 27, 28], while others reported that the outcome of surgery plus RT were similar to those of definitive RT alone, but with a higher risk of severe complications [19, 25, 29]. We therefore first performed a comparison of RT alone with RT plus surgery. The pooled analysis demonstrated that RT plus surgery was associated with a greater 5-year OS rate than RT alone. Moreover, there was a beneficial trend toward a higher 5-year DFS, though the effect was not significant. The higher CR rate and lower NR rate is also consistent with a survival benefit from the combination of surgery and RT.

For neck-irradiation settings, the current guidelines suggest treating the involved lymph node field [34]. However, some reports indicate that patients administered RT to the bilateral neck nodes appeared to have greater local control and higher survival rates than those who received only ipsilateral irradiation [1, 4, 6, 15]. In the present pooled analysis, significantly less contralateral cervical recurrence or emergence of a primary tumor was noted in patients receiving bilateral irradiation, and there was trend toward increased 5-year OS and DFS. These findings suggest that current guidelines recommending the involved lymph node field as the standard RT schedule may need to be re-evaluated for the NCUP setting.

As to the value of irradiation of the PPTS, although current guidelines recommend it as routine consideration for inclusion in the target volume [34], conclusions drawn from currently available evidence are controversial. Some studies have shown a higher 5-year OS rate and better regional control with addition of irradiation of potential head and neck mucosal sites of cancer growth [1, 4–6, 8, 13, 15], whereas, other trials observed that mucosal irradiation reduced both the emergence of primary tumors and regional recurrence, but did not affect OS [3, 7, 12]. In the pooled analysis of all these trials, not only was an advantage for regional control validated, so was a survival benefit in patients treated with irradiation of the neck and PPTS.

We also evaluated the toxic effects of different RT regimens. Because the data were limited, we only assessed severe acute toxicities and xerostomia. We found a significantly higher risk of these adverse events in the RT to the bilateral neck plus PPTS group. However, it is believed that these severe acute toxicities are clinically manageable, and xerostomia could be minimized by application of intensity modulated RT (IMRT) [11, 15].

There were several limitations to this study. First, all the included studies were retrospective and the sample groups were small. There was not sufficient data to perform subgroup analyses based on lymph node levels, lymph node stages, histological types, sequence of surgery and RT, or radiation dosage. Second, these studies were performed over a long time-span. Consequently, the techniques for delivering RT were varied, and precision RT techniques, such as 3-D conformal RT and IMRT, were not yet broadly applied. This could result in an underestimation of the actuarial effect of RT. To address these issues, future multicenter RCTs are needed.

## MATERIALS AND METHODS

### Literature search and selection

Two authors (X.M.L. and X.X.Z.) independently carried out systematic literature searches of EMBASE, Cochrane, Pubmed and Web of Science before September 25, 2015. The following terms were used: occult primary, unknown primary, neck lymph node, cervical lymph node, metastatic, metastases, cancer, neoplasm, tumor, carcinoma, radiotherapy, irradiation, radiation.

Studies meeting the following selection criteria were included. (1) Study population: patients with cervical lymph node metastases from unknown primary sites, and with no cancer history. (2) Study design: comparative studies comparing RT alone with a combination of RT and surgery (radical neck dissection, selective neck dissection, or excisional biopsy); comparing ipsilateral irradiation with bilateral irradiation; or comparing neck-only irradiation with neck and potential primary tumor site (PPTS) (nasopharynx, oropharynx, larynx, and/or hypopharynx) irradiation. (3) Language: English. (4) Studies with available data on at least one of the pre-specified endpoints: 5-year OS, 5-year disease free survival (DFS), neck recurrence (NR), complete response (CR), primary tumor emergence (PTE), ipsilateral neck recurrence (INR), contralateral neck recurrence (CNR), severe acute toxicity (RTOG grade≥3) and xerostomia. Editorials, letters to the editor, and review articles were excluded (Figure 1).

### Data extraction

The following items were extracted independently by the two authors (X.M.L. and D.H.L.) from the published articles: year of publication, first author, country, study period, demographic and clinical information on the study patients (age, gender, histology, N stage, N level), schedule of treatment, number of patients, outcome results, and follow-up. Any disagreement was resolved through further discussion and including a third author.

### Quality assessment

The quality of each study was assessed using the Newcastle-Ottawa Quality Assessment Scale (NOS), which considers of the following factors: patient selection, comparability of the study groups, and assessment of outcome [35, 36]. Each study was assigned a score of between 0 and 9. Any discrepancies were resolved by discussion. Studies with a score of > 5 were regarded as high-quality studies.

### Statistical analysis

All the meta-analyses were performed using Review Manager 5.2 (Cochrane Collaboration, Oxford, UK). Each pre-specified outcome was measured in terms of the risk ratios (RRs) [36] and its 95% confidence intervals (CIs). Two-sided values of *P* < 0.05 were considered statistically significant. Heterogeneity among studies was determined by the Chi-square test and an inconsistency (I^2^) statistic of forest plots. I^2^ > 40% or *P* < 0.10 indicated significant heterogeneity [37]. If there was significant heterogeneity among studies, a random-effects model was used. Otherwise, a fixed-effects model was used [37, 38].

## CONCLUSIONS

This study suggests that, in patients with NCUP, surgery combined with RT to bilateral neck and PPTS may be the preferable treatment option, as it is associated with improvements in survival and regional control. On the other hand, this recommendation is not based on randomized trials, and one must be alert for severe acute toxicity and xerostomia.

## SUPPLEMENTARY MATERIALS


