# Supplementary material for: Optimization of radiotherapy for neck carcinoma metastasis from unknown primary sites: a meta-analysis
Source: Oncotarget. 2016 Oct 24;7(48):78736–46. doi: 10.18632/oncotarget.12852 (PMC5346673; doi:10.18632/oncotarget.12852)
Supplement: Supplementary file 1 [file oncotarget-07-78736-s001.pdf]

Optimization of radiotherapy for neck carcinoma metastasis from unknown primary sites: a meta-analysis

Supplementary Materials

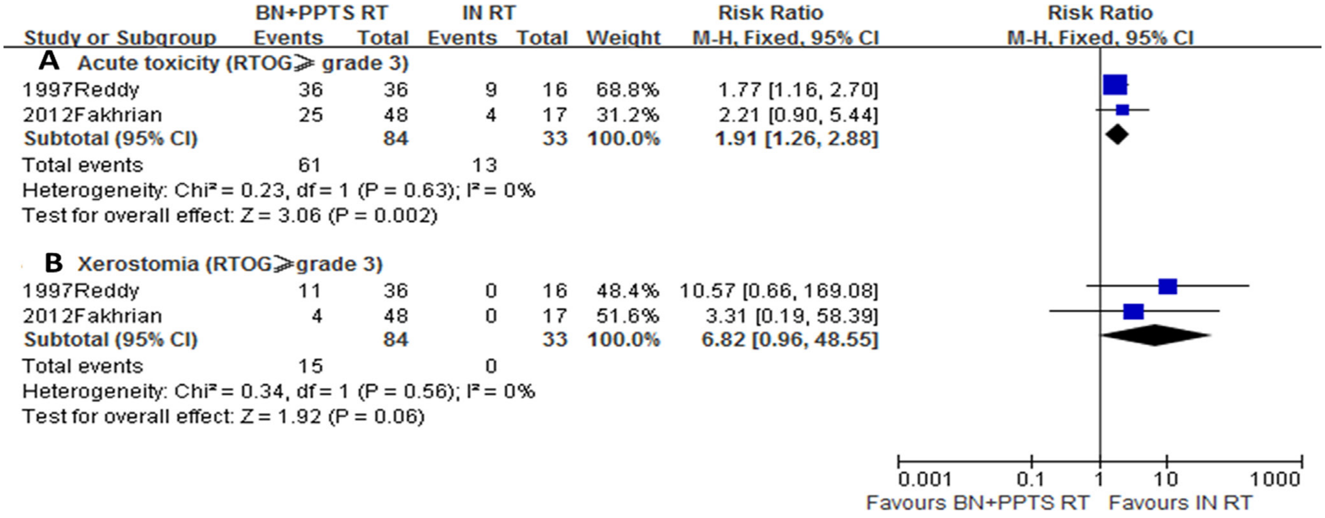

Supplementary Figure S1: Meta-analysis of incidence of (A) acute toxicity (RTOG grade ≥ 3) and (B) xerostomia (RTOG grade ≥ 3) associated with different RT regimens. RTOG, Radiation Therapy Oncology Group; IN, Ipsilateral neck; BN+PPTS, Bilateral neck plus potential primary mucosa sites; RT, Radiotherapy.
